# Effects of a nutrition education intervention on food-provision managerial decisions in Catalan old age nursing homes

**DOI:** 10.1371/journal.pone.0310856

**Published:** 2024-12-16

**Authors:** Beatriz Rodríguez-Sánchez, Isaac Aranda-Reneo, Toni Mora

**Affiliations:** 1 Department of Applied Economics, Public Economics and Political Economy, Faculty of Law, University Complutense of Madrid, Madrid, Spain; 2 CIBERFES, ISCIII, Spain; 3 Economic and Finance Department, Faculty of Social Sciences, University of Castilla-La Mancha, Talavera de la Reina, Spain; 4 Research Institute for Evaluation and Public Policies (IRAPP), Universitat Internacional de Catalunya, Barcelona, Spain; University of Georgia, UNITED STATES OF AMERICA

## Abstract

**Introduction:**

Existing research does not provide definitive conclusions on the most effective initiatives for preventing malnutrition among older adult residents in nursing homes.

**Objective:**

We aimed to assess whether a nutrition education intervention provided to nursing home managers can improve dietary managerial decisions within nursing homes.

**Methods:**

We performed a multicenter study, where each center was randomized to an intervention or a control group. To ensure homogeneous group representation, we stratified the sample based on the facility size and the availability of kitchen equipment. The baseline survey contained questions related to center characteristics, kitchen size and availability of cooking tools, availability of different daily menu options and the staff working in the kitchen. The follow-up survey included questions about the staff responsible for making nutritional decisions, the degree of food handling, the availability of texture-modification tools, and the residents’ satisfaction. We use ordered probit regression models to estimate the effect of the educational intervention on decisions around food management.

**Results:**

From the 238 nursing homes that responded to the initial survey, 176 were followed-up; 56 were allocated to the intervention group and 120 to the control group. There were 53.32 residents per center among the treated group and 40.82 residents in the non-treated institutions. The intervention increased by 24% the nursing homes’ probability of increasing their stock of texture-modification tools, which rose to 26% after controlling for the effect of receiving specific training on textures or presentation, compared to the control group.

**Conclusions:**

The intervention improved managerial decisions in nursing homes concerning meal presentation through an increase in the number of cooking tools. Incorporating structured and comprehensive sessions to improve food texture and presentation could help fight the risk of residents’ malnutrition.

## Introduction

Older adults are a growing segment of the population in most countries, and malnutrition within this group has become a concern. While malnutrition may be present before disease manifestation, it is frequently unrecognized [1], resulting in worse health outcomes and higher healthcare costs among older adults in the community [2]. The prevalence of malnutrition was estimated to be 3.3% among older adults living at home and 7.7% among those institutionalized at the beginning of the millennium in Spain [3]. Ten years later, De La Montana and Miguez showed higher malnutrition rates among older adults, reaching 12.5% in people living at home and 57.5% among those institutionalized [4].

Hamirudin et al. provided a systematic review of the factors associated with malnutrition, highlighting the importance of timely nutrition screenings and appropriate intervention and monitoring [5]. Previous epidemiological research demonstrated that, among older adults, weight loss is associated with an increased risk of mortality, higher hospitalization rates [6] and, consequently, with higher drug consumption. Oropharyngeal dysphagia is a common cause of malnutrition and a significant healthcare concern, especially in nursing homes [7, 8]. While ample literature supports the negative impact of malnutrition on different clinical, functional, and economic outcomes for hospitalized patients [9, 10], little information exists about the associated risk factors of malnutrition among older adults living in nursing homes.

Existing research does not provide definitive conclusions on the most effective initiatives for preventing malnutrition among older adults [11], especially among those who are functionally limited in some activities of their daily living, such as preparing meals or feeding themselves, such as nursing home residents are. Previous studies addressing malnutrition among older adults have primarily focused on the characteristics of the residents, informal caregivers, and non-clinical community care workers [12], along with aspects such as food improvement and staff training [11, 13]. A study on institutional settings in the United Kingdom concluded that the way in which meals are served and presented, and the environment where meals are provided, are important organizational factors in maintaining adequate nutrition among residents [14, 15]. Divert et al. stated that changing even a single contextual meal element could significantly improve the residents’ satisfaction, increase their portion size consumed, and considerably reduce the risk of malnutrition [16]. More evidence on managing malnutrition in nursing homes is needed since research on meal presentation, cooking methods and implements, and kitchen issues is scarce.

This study aims to gain insight into nutrition education by assessing the impact of an intervention (nutrition workshops) that target nursing home managers to improve the food intake of residents, compared to care homes in the control group, who provided services without the benefit of the information provided at the workshops. The analysis was performed at care-home level, with data collected using surveys before and after the nutrition workshops were held.

## Methods

### Study design

This was a multicenter study, where each center was randomized into either treatment or control: three intervention arms and one control group. We compared the results obtained in the intervention group with the control group, which received no intervention and continued to provide their standard care. To ensure homogeneous group representation, nursing homes were stratified based on the facility size and the availability of kitchen implements, with no exclusion criteria required. Inclusion criteria was to be a nursing home placed in Catalonia (Spain) and the randomized unit was the nursing home using a simple randomization method (all nursing homes were numbered and, using Stata, random numbers were assigned to each group).

The intervention program consisted of educational sessions. These were undertaken from 15–30 September 2019, with the attendance of up to two staff participants per center, often including the nursing home managers or other senior nursing home staff. Educational sessions focused on guiding nursing and caregiving staff, resident cooks, and the directors of nursing homes. Each session consisted of small groups of 15–25 staff participants from multiple nursing homes receiving a three-hour educational training session by several expert nutritionists from the Alicia Foundation (https://www.alicia.cat/en/), a research center devoted to technological innovation in cuisine, improving eating habits, and evaluating food and cooking heritage. After a one-hour theoretical lecture, two hours were allocated to practical lessons on aspects including meal planning, ingredients, food texture and presentation and other related issues (food temperature, colors, quality, and quantity). The sessions on presentation provided more emphasis on colors and how to present meals. The texture sessions reinforced how to provide the most suitable texture-modified diets and techniques of dysphagia assessment. It should be noted that the sessions were conducted at the Alicia Foundation, located 62 km from Barcelona city, which challenged the participation of some centers. To improve the involvement of centers in the intervention groups that could not attend physical sessions, video recordings of their specific sessions were sent to them. We also provided participating centers with hard copy supplementary materials from the Health Department to help nursing home staff improve their managerial decisions as supporting material after the sessions had taken place. A follow-up survey to assess the intervention impact was planned four months after the baseline survey, but because of the SARS-COV-2 outbreak was postponed until April 2021.

The control group received no material or educational sessions. Thus, all nursing homes allocated to the control group made their own decisions on how to produce and present meals as they had done previously. This trial was not an open-label trial, so none of the participants knew whether they were in the intervention or control group. We asked participants to avoid communicating the contents of the educational session and held each group session on a different date.

The study adhered to the Declaration of Helsinki and was approved by the Ethics Committee of Universitat Internacional de Catalunya (FCES-2018-01).

### Data collection

The list of all nursing homes in Catalonia was obtained from the Public Health Department of the regional government of Catalonia (Generalitat de Catalonia) by sending a letter to the Department of Social Welfare and the Ministry of Health in Spain. Each center was contacted to invite them to participate, and those nursing home managers who agreed provided written informed consent.

A purpose-developed survey was administered online (a pdf version was available) between February and June 2019 to all participating centers. Facilities were classified based on the participants’ responses regarding facility size (number of residents in quartiles) and available kitchen tools (kitchen robots and mixers). The baseline survey contained 31 questions related to four main aspects, mostly by means of closed questions: (i) center characteristics, including the number of residents, the number of chefs and staff responsible for providing food and supplements, and the presence/absence of dietitian support; (ii) the kitchen size and availability of cooking tools; (iii) the availability of different daily menu options, the degree of food modification (categorized into high (own kitchen), moderate (both catering and preparation of garnishments or other dishes), or low (full catering)) and the staff’s prior participation in nutrition courses on texture-modified diets. Moreover, some characteristics of the residents were collected, such as the dependence level (based on the Department of Social Services criteria, the dependence degree was classified as I (mild), II (moderate), or III (high)); nutritional status, classified following the Mini Nutritional Assessment scale as normal nutritional status or at risk of malnutrition or malnutrition [17] and body mass index [BMI]) categories (underweight, normal weight, overweight and obesity) [18], need of help (full, partial) when feeding themselves, the number of meals taken per day at the nursing home (options: breakfast, lunch, snack, dinner) and the incidence of diseases among current residents during the previous thirty days (acute respiratory diseases or psychological stress; pressure ulcers; vascular ulcers; ulcers due to moisture; chronic diarrhea; constipation and vomiting). This survey was developed considering existing literature, personal interviews, and focus groups with participating nursing homes and day-care centers.

The follow-up survey included seven questions regarding the effects of the intervention. Questions were asked about the availability of chefs and dieticians, the staff responsible for making nutritional decisions, the degree of food handling, the availability of texture-modification tools, and the level of satisfaction with food textures and appearance. Closed questions were asked (yes/no). We used this information to compare the baseline levels and assess the intervention’s effect on decisions concerning food management. Managerial decisions around food could refer to: i) whether the right staff were responsible for providing food or supplements, ii) specifically whether there were in-house dietitians, and ii) whether there was a change in the number of implements available for texture modification. The interaction could impact firstly, by changing some or all of the three outcomes mentioned above; or secondly, between baseline and follow-up i) by improving/reducing the degree to which appropriate people led the administration of food and supplements; ii) whether the number of a new in-house dietitians increased/declined; or iii) whether the number of kitchen tools (robot, mixer or both) increased/declined. We considered the intervention led to an improvement (worsening) if the metrics mentioned above increased (declined) between baseline and follow-up.

### Statistical analyses

Nursing home characteristics were analyzed descriptively at baseline, assessing whether there were statistically significant differences between the conditional means in the intervention and control groups (that is, controlling for the mean difference of all the variables simultaneously) using a logistic regression.

We performed an ordered probit regression based on the nature of the endogenous variables (worsened, no change, or improved managerial decisions concerning food-related issues) to estimate the effects attributable to the intervention, accounting for the characteristics at the baseline year. The following empirical model was used:

$$prob_{i}(improvement_{j})=\Phi (\alpha_{i}+\delta_{j}E_{i}+\eta_{j}E_{i}+\beta_{j}x_{i}^{\prime }+\alpha_{i}+\varepsilon_{i})-\Phi (\alpha_{i}+\delta_{j-1}E_{i}+\eta_{j-1}E_{i}+\beta_{j-1}x_{i}^{\prime }+\alpha_{i}+\varepsilon_{i})$$ (1)

$$j=2,\ldots ,j-1prob_{i}(improvement_{j})=1-\sum_{j=1}^{J-1}prob_{i}(improvement_{j})$$

where $prob_{i}(improvement_{j})$ = 1 if nursing home i during time t (t = 0 before the intervention and t = 1 after the intervention) experienced any improvement in the considered outcomes related to dietary decisions (including the availability of texture-modification tools and the dieticians’ role in the nursing homes). E represents a dummy variable that equals 1 for the treatment group; x_i_ represents the changes in the characteristics of the nursing home (facility size and available kitchen tools); α_i_ represents the unobserved fixed effects for nursing homes, and ε_i_ is the random noise, which is assumed to be independent of the explanatory variables. The main parameter of interest is δ—the effects of the nutrition workshops. Standard errors were corrected. Results were assessed on the intention-to-treat basis. Concerning the nursing home characteristics, although our interest lies in the impact of the treatment, we also included the following variables that might impact managerial decisions on nutrition-related choices and might also affect basal differential characteristics when randomizing: (i) variables identifying the percentage of users with the list of diseases mentioned in the “data collection” section above; (ii) change in the number of residents between the baseline and follow-up periods; (iii) previous percentage of residents who required full help to be fed; (iv) previous nutritional training received by the staff; (v) records of patients’ food intake; (vi) time passed between the two surveys; (vii) the percentage of residents affected by SARS-COV-2. These variables would help to correct possible biases due to systematic differences in nursing homes. Moreover, the predicted probability of falling into one of the arms (treatment or control) was used as an instrumental variable. We also computed the marginal effects for those nursing homes that participated in on-site workshops and those who received online training by including the distance from the nursing home to the workshop location as a second instrument, as distance may have conditioned participation. We measured the effect of each type of workshop by using simultaneous equations to jointly estimate all treatments. Statistical analyses were performed using STATA software, version 17.0 (STATA Corporation, College Station, TX, USA).

## Results

**Fig 1:**
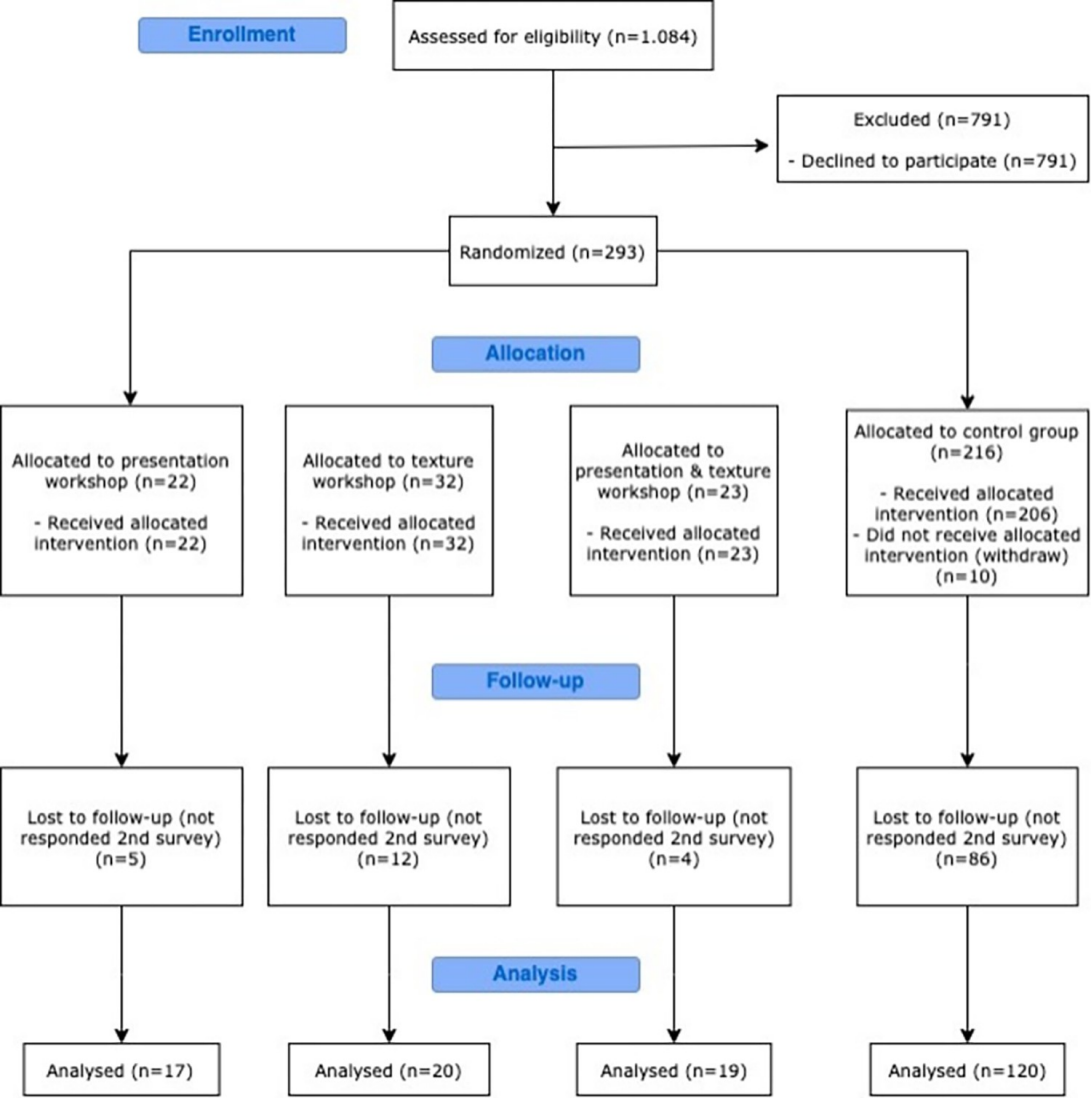
Flow diagram of study participants.

In this study, 293 out of the 1,084 nursing homes approached (response rate, 27%) agreed to participate. Participants from 57 of these facilities attended on-site nutrition education sessions, and 20 attended online, resulting in 77 facilities (22 participated in presentation workshops, 32 in sessions relating to the adoption of texture-modified diets, and 23 in both). Fig 1 shows the enrolled participants’ flow diagram from the study’s beginning until follow-up. The follow-up survey was answered by 176 nursing homes (60.1% of our primary respondents) from September 2020 to April 2021.

Most questions were answered by managers (61.1%), followed by caregivers (29.4%). Descriptive results indicate that the average per-center population was 53.32 residents in the treated group and 40.82 residents in non-treated institutions. The mean number of chefs per center was 2.15 and 1.45, respectively, which is a statistically significant difference. We observed that 32.14% of treated nursing homes reported a lack of systematic registration of the residents’ food intake, compared to 41.67% in the comparator (Table 1). Table 1 shows the main characteristics of nursing homes regarding their internal food management policies and other features by treatment group.

**Table 1:** Nursing homes and day-care centers’ baseline characteristics.

	Treated	Not treated	Logit (p-value)
	N = 56	N = 120	
Nursing home residents	53.32 (39.39)	40.82 (33.50)	0.710
Nutrition-related characteristics of nursing homes			
Number of chefs	2.15 (1.61)	1.45 (1.33)	0.051
Menu selection option	41.07%	33.33%	0.635
Systematic record of residents’ food intake	67.86%	58.33%	0.074
Staff responsible for providing food			
Dietitian	44.64%	34.17%	0.573
Nurse	39.29%	50.83%	0.231
Physician	8.93%	7.50%	0.059
Others (auxiliary, cook, manager)	7.14%	7.50%	0.122
Staff responsible for providing supplements			
Dietitian	12.99%	14.56%	0.152
Nurse	54.55%	64.08%	0.977
Physician	31.17%	16.99%	0.282
Others (auxiliary, cook, manager)	1.30%	4.37%	0.515
Dietitian support			
In-house dietician	8.93%	26.67%	0.358
Externally designed menus	26.79%	6.67%	0.021**
External consultant	42.86%	59.17%	0.362
No dietitian	21.43%	7.50%	0.292
Degree of food modification			
None or low	19.64%	27.50%	
Moderate or high	80.36%	72.50%	0.898
Tools for texture modification			
Robot	14.29%	9.17%	0.882
Mixer	76.79%	81.67%	0.494
Neither robot nor mixer	8.93%	9.17%	0.758
Previous training			
Food presentation	3.57%	11.67%	0.058
Food textures	44.64%	29.17%	0.278
None	51.79%	59.17%	0.232
Characteristics of nursing home residents			
Level of dependence			
Low (I)	18.75%	24.62%	0.295
Medium (II)	42.37%	38.94%	0.069
High (III)	26.08%	27.85%	0.125
Nutritional status			
Malnutrition	12.74%	5.14%	0.168
Risk of malnutrition	17.91%	17.99%	0.176
Normal nutritional status	69.35%	76.87%	0.166
Dysphagia prevalence	13.58%	11.84%	0.948
BMI			
BMI < 19 kg/m^2^	9.04%	10.05%	0.134
19 kg/m^2^ ≤ BMI < 23 kg/m^2^	34.39%	32.29%	0.319
23 kg/m^2^ ≤ BMI < 27 kg/m^2^	36.06%	36.59%	0.637
BMI ≥ 27 kg/m^2^	20.75%	21.08%	0.148

Note: Data are expressed as mean (Standard deviation) or %; ** p-value < 0.05.

Food and supplements were provided mainly by dietitians and nurses. Physicians also prescribed supplements and occasionally food. However, when conditional on all the other characteristics, these means were non-significantly different in terms of differences between the two groups. Considering dietitian support, 8.93% was provided by in-house dietitians in the treatment group versus 26.67% in the standard-of-care group. These differences became more significant in the case of externally designed menus (26.79% vs. 6.67%). Regarding tools available for modifying food textures, 14.29% of the facilities had a kitchen robot, 76.79% only had a mixer, and 8.93% did not have any of these items in the treated nursing homes, with none of these differences being significantly significant (Table 1). 81.67% of the institutions in the control group had a mixer, and 9.17% had a robot. In both groups, more than half of the participating nursing homes had not previously been given training, whereas 44.64% of the staff in the treated nursing homes had already received food-texture training (compared to 29.17% in the control group).

Concerning the residents’ characteristics in the treated nursing homes, 12.74% had malnutrition, 17.91% were at risk, and 69.35% were well-nourished. In contrast, those figures were 5.14%, 17.99%, and 76.87%, respectively, in the control group (Table 1). Still, these differences were not significant when conditional on the mean of all the listed variables in the table. Only 9.04% of the residents in the treated group showed a BMI below 19 kg/m2 and 20.75% presented a BMI above 27 kg/m2. Similar figures were found in the control group. The percentage of residents in treated nursing homes who showed a medium level of dependence (42.37%) was significantly higher than those observed in the control group (38.94%).

The residents’ ability to feed themselves is crucial in preventing weight loss and malnutrition. Although there was a considerable variation at the nursing home level at baseline, the variation in the percentage of residents requiring partial help (see S1 Fig panel b) was more variable across nursing homes than those requiring full assistance (see S1 Fig panel a), for which most homes saw levels below 30% of their residents.

Table 2 shows the specific changes concerning the staff responsible for diet between baseline and follow-up at treated and controlled nursing homes. The upper part of the table, which refers to the treated nursing homes, shows that the main transfer in terms of diet responsibility was from dietitians to nurses. Specifically, of the 25 dietitians responsible at baseline, 14 of them were replaced by nurses (more than 50%) at follow-up. The lower part of the table reports the same transition between dietitians and nurses in the control group. However, the percentage is not as high as in the treatment group (35% of dietitians were replaced by nurses by the follow-up survey).

**Table 2:** Transitions in the staff responsible for diet control from baseline (rows) to follow-up (columns).

	**Treated nursing homes**				
*Baseline / Follow-up*	**Dietitian**	**Nurse**	**Medical doctor**	**Geriatric assistant**	**Cook**
Dietitian	11	14	0	0	0
Nurse	8	15	0	0	1
Medical doctor	3	1	2	0	0
Geriatric assistant	0	0	0	0	0
Cook	1	0	0	0	0
	**Control nursing homes**				
*Baseline / Follow-up*	**Dietitian**	**Nurse**	**Medical doctor**	**Geriatric assistant**	**Cook**
Dietitian	20	19	4	1	1
Nurse	11	44	0	0	1
Medical doctor	3	1	3	0	0
Geriatric assistant	0	1	0	0	0
Manager	1	1	0	0	0

**Fig 2:**
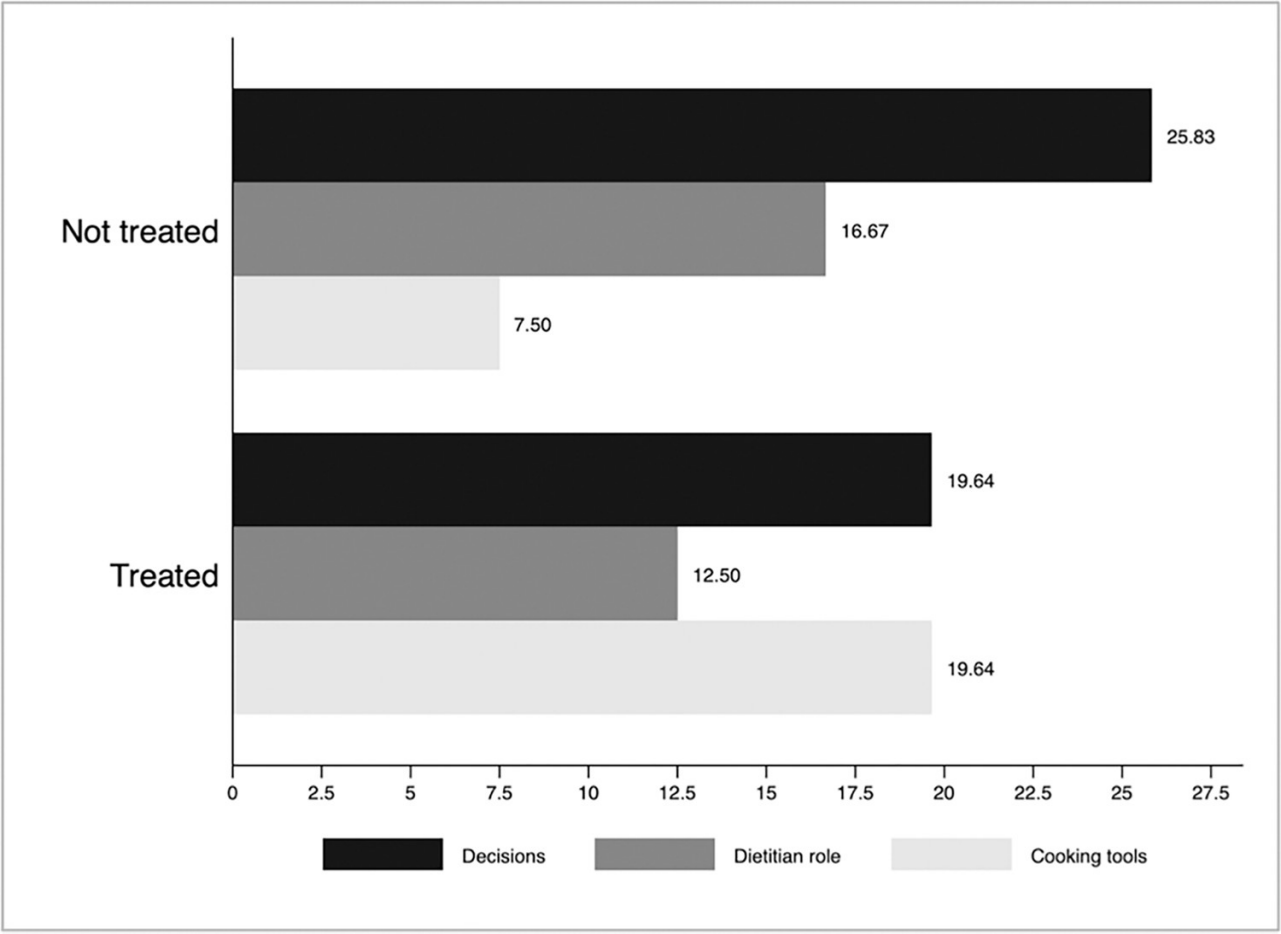
The proportion of nursing homes with improved outcomes by treatment group at the end of follow-up. Note: bar graphs show the proportion of nursing homes showing improvement in dietary decisions, dietitians’ role, modification of food texture, and availability of cooking tools for modifying textures.

Nursing homes in the intervention group improved availability of texture-modification tools between baseline and follow-up by more than those in the control group (19.6% vs. 7.5%). However, the control group showed more significant improvements in nutrition-based decisions and the dietitian’s role (Fig 2).

An empirical approach was performed to account for the characteristics at the baseline year by reporting the marginal effects of the intervention for each considered decision and outcome (ordinal). The impact of nutrition workshops was significant in improving the availability of texture-modification tools, with a 23.8% increase in the intervention group, compared to the control group, with no statistically significant effect of the nutritional education intervention being observed for the other managerial decisions assessed (Table 3). These results were consistent after introducing inverse probability weights that accounted for attrition. No pattern of attrition was observed based on observables.

**Table 3:** Marginal effects of the intervention: Instrumental variables estimation.

	Nutritional decisions	Dietitians’ role	Availability of cooking tools
*Treatment group* ^*a*^			
No variation	0.022 (0.03)	-0.015 (0.02)	-0.143 (0.07) **
Improvement/Increase	0.105 (0.14)	0.099 (0.13)	0.238 (0.09) ***
*Wald Chi*^*2*^ *(p-value)*	186.46 (0.00)	157.69 (0.00)	159.29 (0.00)

Note: The sample size was 176 nursing homes.

*** significant at 1%

**significant at 5%

*significant at 10%. The partial R^2^ for the first regression was 0.411, F = 121.8 (0.00). The intention-to-treat parameter was 0.412 (p-value = 0.00). All regressions included the following covariates: (i) variables identifying the percentage of users with several diseases (acute respiratory, pressure and vascular ulcers, moisture lesions, chronic diarrhea, nausea, constipation, and others); (ii) changes in the number of residents between the baseline and follow-up survey; (iii) percentage of residents who required full help to be fed; (iv) staff with previous nutritional training; (v) systematic record of food intake; (vi) time between the two surveys, (vii) percentage of residents affected by SARS-COV-2; and, (viii) percentages accounting for the number of residents having different meals (breakfast, lunch, afternoon snack and dinner). The estimation also accounted for the initial conditions for the outcomes.

^a^ The interaction could impact firstly, by changing some or all of the three outcomes mentioned above; or secondly, between baseline and follow-up i) by improving/reducing the degree to which appropriate people led the administration of food and supplements; ii) whether the number of in-house dietitians increased/declined; or iii) whether the number of kitchen tools (robot, mixer or both) increased/declined.

We considered the intervention led to an improvement (worsening) if the metrics mentioned above increased (declined) between baseline and follow-up.

When analyzing the results for those centers with on-site participation in the workshops, we observed that the significant marginal impact increased to 38%. The marginal effect was not effective for those who received a workshop on food presentation but for those who attended workshops on food textures or both textures and presentation, the availability of texture-modification tools increased by 13.5% and 26.3%, respectively. Both coefficients were statistically significant at 1% levels for the randomized groups.

## Discussion

This study analyzed the impact of nutrition workshops (food presentation, adoption of texture-modified diets, or a combination of both) on factors relating to the presentation and texture of the food served at nursing homes in Catalonia. Our results showed that providing training to the nursing home staff led to a 24% increase in the availability of texture-modification tools, and on-site participation in the workshop increased its availability to 38%, compared to nursing homes that did not receive the assessed educational nutrition intervention. Studies have shown that improving food presentation and texture significantly impacts residents’ food acceptance [19] and leads to higher customer satisfaction and avoidance of adverse events [16], such as malnutrition. Therefore, the intervention assessed in this study could be an effective approach to improving residents’ food acceptance and, hence, food intake, which could eventually result in a reduction of the prevalence of malnutrition among nursing homes residents.

Descriptive data on nursing home internal policies revealed a lack of systematic registration of residents’ food intake in 32% of the treated facilities and more than 40% of the nursing homes in the control group. Moreover, 21.43% and 7.50%, respectively, did not have dietitian support at baseline. These figures reinforce the importance of promoting interventions that can help to improve the management of residents’ nutrition. Notably, malnutrition levels (12.74% in the treatment group and 5.14% in the control nursing homes) aligned with data from previous Spanish studies [5, 6], whereas the risk of malnutrition seemed slightly lower than expected. The latter might be related to the percentage of residents with high or medium levels of dependence in the nursing homes included (26.08% and 42.37%, respectively, in the treated nursing homes’ residents, and 27.85% and 38.94%, respectively, in the control group). Previous studies have shown that the setting, in this case, nursing homes, might determine the prevalence of malnutrition [20, 21], as well as identifying a positive relationship between malnutrition and some geriatric syndromes, such as functional dependence [22–24].

One of the most important results of our study was that providing specific nutrition education made nursing home managers more likely to acquire tools for improving the texture and appearance of the food served to the residents. The percentage of homes in the intervention group who utilized such tools increased by 24% between baseline and follow-up, compared to the control group. Since there is evidence that texture-modified food may help dysphagic residents and those with physiological behaviors and bolus flow patterns [19], we would expect that the intervention will improve residents’ health.

Another relevant result was the change in the staff responsible for diet control. That role was shared between dietitians and nurses among the centers allocated to the intervention group, whereas in the control group, nurses were most likely to take charge. This suggests that nursing home managers were influenced by the intervention to the extent that they decided change was needed. Despite this, when adjusting for additional features in the regression model, the results showed that the intervention did not play a significant role in determining the different nutritional decisions or dietitians’ roles in treated versus control-group nursing homes.

### Limitations and implications

This study has some limitations that are worth considering. It was impossible to assess the nutrition status of residents and related problems due to pandemic restrictions, as well as to collect any other information at the residents’ level. The initial idea was to perform the study at the individual level, the resident, but after the COVID-19 outbreak, the only possibility to run the study, as agreed with the corresponding institutional body, was to perform it at the nursing home level. The promising result identified in the study was observed with the intervention despite the constraints imposed by the SARS-COV-2 pandemic, which had a huge impact on nursing homes in all countries, particularly in Spain [25, 26]. Based on official data [27], around 20,200 residents– 47–50% of Spanish nursing home residents–died during the initial outbreak of the SARS-COV-2 pandemic. Although the intervention was implemented in October 2019, the pandemic led us to postpone the follow-up survey until the end of April 2021. Therefore, in the statistical analysis, we included the time passed between baseline and follow-up as a way of identifying possible differences between facilities. Additionally, day-care centers were forbidden to operate during the first wave of the pandemic (March 2020 –May 2020), which prevented us from following up with the centers in that category that participated during the baseline year. This led to the removal of day-care centers from the study and the results reported in this article. Although the data collected on nursing homes and day-care centers are similar at baseline, adding post-intervention information from day-care centers could have affected the applicability of results. Nevertheless, delays caused by the pandemic allowed us to observe the effects of the intervention over a more extended period and in a particularly complicated situation. In this regard, the 60.1% response rate of the follow-up survey constitutes a success considering the situation. However, attrition issues still had an impact on the study, although inverse probability weights were used to account for this issue. The 24% increase in the availability of texture-modification cooking tools observed after controlling for the time difference between the assessments is promising and unexpected. The pandemic’s impact likely worsened the previously observed characteristics and resources available to nursing homes. This is because nursing homes worst affected by the pandemic would have had less flexibility when making decisions on aspects such as nutritional choices. There are other factors outside the scope of this study that could have been relevant to improving residents’ food intake or related to it. These include changes in residents’ nutritional status, which was only assessed and recorded during the baseline data collection. While this study assessed the availability of texture-modification tools, results on the improvement of food presentation were not discussed, although this variable might be measured with bias. Actually, for future studies, we may consider including a more objective measure to assess the effect of modified food textures, such as the International Dysphagia Diet Standardization Initiative (IDDSI). The IDDSI has a standard classification system for the classification of texture modified foods to address dysphagia [28]. Finally, we conducted a not open-label study clustering participants based on their location to avoid information crowding out and switched their initial assignment if the facility belonged to the same institution. These considerations should have helped to minimize the presence of bias.

Despite these limitations, this study is one of the largest assessing a nutritional education intervention targeting nursing home managers in Spain. The efficacy of this intervention encourages us in post-pandemic times to propose nutrition education as a new policy program of the health department to improve the service provided by nursing homes.

Those making managerial decisions can benefit from the current evidence reported in this article. The evidence can help decision-makers in nursing homes to improve food presentation and textures which should lead these facilities to reductions in malnutrition prevalence, avoidance of adverse events, and higher customer satisfaction, as the existing evidence has already demonstrated. The educational sessions assessed in the present study have demonstrated that increasing the availability of texture-modification tools is one of the mechanisms for better food presentation. Moreover, helping decision-makers with non-clinical issues, such as food presentation, encouraged them to increase the use of dietitians. Incorporating such health professionals in collaboration with nurses might lead to the formation of multidisciplinary teams who can provide facility residents with better assistance. Further studies that analyze this effect in the medium and long term are needed.

## Conclusions

This study shows that educational sessions focused on presentation and food texture medication provided to nursing home staff can lead to the provision of better services and improved decision-making in nursing homes. This is likely to reduce rates of malnutrition amongst residents and consequently better health. We are encouraged that our study finds positive results, even during the complex situation caused by the SARS-COV-2 pandemic. We suggest that further studies are required to consider the cost-effectiveness elements of such interventions. Additionally, we propose that adding elements around nutrition to nursing staffs’ on-the-job training could prove valuable.

## Supporting information

S1 Fig The proportion of patients requiring full or partial help for regular food intake. Graphs show the frequency distribution of residents requiring full (a) or partial (b) help for regular food intake at baseline.
(DOCX)
