# Supplementary material for: Effects of a nutrition education intervention on food-provision managerial decisions in Catalan old age nursing homes
Source: PLoS One. 2024 Dec 16;19(12):e0310856. doi: 10.1371/journal.pone.0310856 (PMC11649126; doi:10.1371/journal.pone.0310856)
Supplement: S1 Fig — Graphs show the frequency distribution of residents requiring full (a) or partial (b) help for regular food intake at baseline. (DOCX) [file pone.0310856.s001.docx]

**Figure 1.** The proportion of patients requiring full or partial help for regular food intake. Graphs show the frequency distribution of residents requiring full (a) or partial (b) help for regular food intake at baseline.

(a)

(b)
